# Role of Osteogenic Growth Peptide (OGP) and OGP(10–14) in Bone Regeneration: A Review

**DOI:** 10.3390/ijms17111885

**Published:** 2016-11-22

**Authors:** Suzane C. Pigossi, Marcell C. Medeiros, Sybele Saska, Joni A. Cirelli, Raquel M. Scarel-Caminaga

**Affiliations:** 1 Department of Diagnosis and Surgery, School of Dentistry at Araraquara, UNESP–São Paulo State University, Humaita St, 1680, CEP 14801-903 Araraquara, São Paulo, Brazil; supigossi@ymail.com (S.C.P.); marcellmedeiros@gmail.com (M.C.M.); cirelli@foar.unesp.br (J.A.C.); 2 Department of Morphology, School of Dentistry, UNESP– São Paulo State University, Humaita St, 1680, CEP 14801-903 Araraquara, São Paulo, Brazil; 3 Department of General and Inorganic Chemistry, Institute of Chemistry, UNESP–São Paulo State University, Professor Francisco Degni St, 55, CEP 14800-900 Araraquara, São Paulo, Brazil; sysaska@gmail.com

**Keywords:** osteogenic growth peptide, bone regeneration, osteoblasts, biocompatible materials

## Abstract

Bone regeneration is a process that involves several molecular mediators, such as growth factors, which directly affect the proliferation, migration and differentiation of bone-related cells. The osteogenic growth peptide (OGP) and its C-terminal pentapeptide OGP(10–14) have been shown to stimulate the proliferation, differentiation, alkaline phosphatase activity and matrix mineralization of osteoblastic lineage cells. However, the exact molecular mechanisms that promote osteoblastic proliferation and differentiation are not completely understood. This review presents the main chemical characteristics of OGP and/or OGP(10–14), and also discusses the potential molecular pathways induced by these growth factors to promote proliferation and differentiation of osteoblasts. Furthermore, since these peptides have been extensively investigated for bone tissue engineering, the clinical applications of these peptides for bone regeneration are discussed.

## 1. Introduction

Interest in developing bioactive biomaterials capable of promoting bone regeneration has grown significantly over the past few years. Different approaches and materials have been developed to regenerate bone defects wherein physiological processes to a bone neoformation are insufficient to repair the injury. In addition, some researchers have focused on investigating biological mediators and the signaling pathways that influence bone metabolism, in order to better understand this complex process. In this context, comprehension of the key signaling pathways involved in bone metabolism is extremely important for the development of bioactive biomaterials related to bone repair or regeneration.

Bone regeneration is a complex process which involves a combination of many biological factors [1]. Growth factors, related to bone repair/regeneration, are biologically active peptide or protein hormones that affect immune functions as well as the proliferation, chemotaxis and differentiation of epithelial, bone and connective tissue cells [2]. These hormones bind to specific cell surface tyrosine kinases receptors, which are present on various target cells including osteoblasts, cementoblasts and fibroblasts [3]. Several growth factors regulate the recruitment and differentiation of bone-related cells. Table 1 summarizes the activity of some of these molecules that are essential in the signaling pathways of the bone remodeling cycle [4]. More details about the main growth factors can be found in the Supplementary Material.

**Figure 1:**
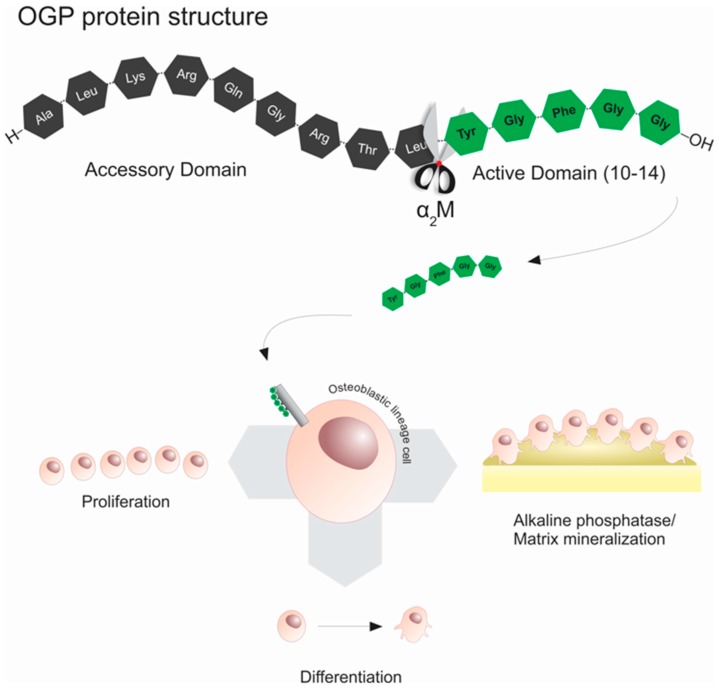
Schematic illustration of the structures of osteogenic growth peptide (OGP) and OGP(10–14) peptides and the interaction of the active domain protein with the osteoblastic lineage cells resulting in increased proliferation, differentiation, alkaline phosphatase activity and matrix mineralization.

Amongst the growth factors related to bone repair/regeneration, an important growth factor named osteogenic growth peptide (OGP) has been widely studied alone or in association with different biomaterials to improve bone regeneration. OGP is a native molecule with a primary structure identical to the C-terminus of histone H4, whose sequence contains a highly conserved 14-amino acid motif (NH_2_-ALKRQGRTLYGFGG-OH). This peptide was isolated from blood during osteogenic remodeling of post-ablation of marrow regeneration [33]. Additionally, OGP peptide when proteolytically cleaved generates the C-terminal pentapeptide (NH_2_-YGFGG-OH), named OGP(10–14) (Figure 1). OGP(10–14) has been considered the physiologically active form of OGP, since this C-terminal sequence activates the cytoplasmic OGP signaling pathway [34], suggesting that OGP(10–14) is the bioactive form of OGP [35,36]. The literature has demonstrated that OGP/OGP(10–14) plays an important role in bone repair/regeneration, mainly in stimulating the proliferation, differentiation, alkaline phosphatase activity and matrix mineralization of osteoblastic lineage cells [33,37]. Furthermore, OGP regulates the expression of Transforming Growth Factors (TGFs), Insulin-like Growth Factors (IGFs) and basic Fibroblast Growth Factors (bFGFs) [38], thus increasing bone formation and trabecular bone density in vivo [39,40].

This literature review approaches the biological key role of OGP and OGP(10–14) and the potential molecular pathways involved in osteoblast proliferation and differentiation, as well as discussing several clinical applications of these peptides for bone repair/regeneration. The methods utilized in the literature review are described in the Supplementary Material. 

## 2. Literature Review

### 2.1. Characteristics of Osteogenic Growth Peptide (OGP) and the Pentapeptide OGP(10–14)

OGP was initially isolated from regenerating rat bone marrow by Bab et al. [33]. Moreover, Bab et al. [41] directly demonstrated the synthesis of OGP from an histone H4 mRNA. OGP is a 14 amino acid peptide identical to the C-terminal residues (90–103) of histone H4. In order for bioengineers and bone biology researchers to better understand this, the translational initiation of OGP occurs at the 85th amino acid (AUG) of the histone H4 gene. This predicts the synthesis of a 19-amino acid H4 peptide (amino acids 85–103), designated preOGP. PreOGP is then converted to OGP by removal of its five amino-terminal residues H4 (90–103). It was suggested that the de novo synthesis of OGP via alternative translation of H4 mRNA facilitates the production and secretion of OGP independent of H4 protein synthesis [41]. This translational initiation at the AUG codon is uncommon for genes encoding structural proteins, but rather frequently observed in genes encoding regulatory polypeptides such as cytokines, receptors, protein kinases, transcription factors and growth factors [42].

OGP has an identical amino acid sequence and immunoreactivity in human and rat sera [43]. The main OGP form in serum is an OGP–OGP binding protein (OGPBP) complex which is physiologically and highly abundant. Changes in the serum levels of bound and unbound OGP to this protein complex, accompany the osteogenic phase of post-ablation bone marrow regeneration and are associated with the systemic osteogenic response [44]. This evolutionary conservation associated with high abundance of circulating OGP suggests potential applications of this peptide for tissue engineering and regenerative medicine. As reported, most of the serum OGP is noncovalently bound to OGPBPs. Gavish et al. [44] suggested a potential role of the α2_-_Macroglobulin (α2M) in this process, as recently outlined in Policastro and Becker [45]. α2M is a major serum multifunctional protein that binds several regulatory polypeptides [46] and has been found in two conformations: a primary circulating form, referred to as the “native” form, and another one as the “activated” form. Both forms of α2M, from human plasma, bind noncovalently to OGP. The α2M appears to be an important modulator of OGP action, since the native protein form significantly enhances OGP osteoblastic mitogenic activity, while the activated α2M form negatively regulates this process [44].

Two other endogenous OGP–OGPBP complexes were identified in murine osteoblastic cells by Greenberg et al. [47], OGPBP-1 and OGPBP-2. They suggested that these complexes have a key role in maintaining large reservoirs of inactive OGP protected from proteolytic degradation and clearance, hence providing a mechanism which controls the availability of the peptide to target cells. Moreover, this study also showed that OGP production is up- and downregulated, respectively, by low and high doses of exogenous OGP in a manner consistent with an autoregulated feedback mechanism [47].

Another important aspect regarding the structural and functional characterization of OGP is the role of the N- and C-terminal regions in OGP–OGPBP complex formation. Greenberg et al. [35] introduced modifications in the OGP terminals and observed that N- and C-terminal-modified analogs displayed a decreased binding activity to the OGPBP. Furthermore, this modification resulted in a decrease in the effect of OGP on the proliferation of osteoblasts and fibroblasts, suggesting a role for the N- and C-terminal regions in the binding of OGP to its putative receptor. Bab et al. [48] demonstrated that the full length OGP and the OGP(10–14) are two active mitogens in the serum, with OGP(10–14) being the physiologically active form of OGP. This pentapeptide is generated from OGP by proteolytic cleavage upon dissociation of the OGP–OGPBP complex.

Considering that the OGP(10–14) is the minimal amino acid sequence that retains the full OGP-like activity, OGP(10–14)-derived peptides have been designed and studied, such as desamino-OGP(10–14) or (daOGP(10–14)), several cyclostereoisomers of OGP(10–14), including the retro-inverso analog (Gly-Gly-d-Phe-Gly-d-Tyr). These OGP(10–14) analogs demonstrated considerable proliferative activity in both osteoblastic or fibroblastic cell cultures, being equipotent or slightly more potent than to OGP-like bioactivities, whereas the manipulations of the carboxyl function at the C-terminus resulted in substantial reduction in mitogenic activity [36,40,49]. In addition, the retro-inverso analog c(Gly-Gly-Phe-Gly-Tyr), a cyclostereoisomer of c(Tyr-Gly-Phe-Gly-Gly), demonstrated to be as potent as the parent cyclic pentapeptide OGP(10–14), and substantially more potent than the linear retro-inverso analog [36]. These studies have also revealed that the pharmacophores present by the side chains of Tyr^10^ and Phe^12^ play a key role in the OGP(10–14) bioactivity, but not sufficient for the complete proliferative activity. Indeed, the residues Tyr^10^, Phe^12^, Gly^13^, and Gly^14^ of C-terminal sequence are essential for the optimal bioactivity of the OGP(10–14) [36,40]. Furthermore, the functionalization of carboxyl termini of the daOGP(10–14) analog by replacing the negatively charged group by neutral ones such as carboxamide, hydroxymethylene or methoxycarbonyl resulted in analogs with 3–4-fold reduced potency compared to that of daOGP(10–14), which is the minimal structure that displays OGP-like in vitro proliferative activity equipotent to OGP(10–14) [40]. Additionally, stepwise truncation from either the N-terminal or C-terminal termini of OGP(10–14) indicated that the mitogenic potency markedly decreased, suggesting a bioactive role for both the N- and C-terminal sequences (Tyr-Gly-Phe and Gly-Gly, respectively), mainly the deletion of either Tyr^10^ or Gly^14^ resulted in considerable loss of bioactivity [40].

In vivo studies indicated better bioactivity effects of the peptides desamino[Tyr^10^]OGP(10–14) and cyclic analog OGP(10–14) in comparison with OGP(10–14). Ovariectomy-induced bone loss in mice model showed that the OGP reverses the majority of trabecular bone loss and that OGP(10–14) and desamino[Tyr^10^]OGP(10–14) have an even stronger osteogenic effect than OGP [40]. Moreover, the OGP(10–14) cyclostereoisomer (Tyr-Gly-Phe-Gly-Gly) was more effective than the less rigid linear OGP(10–14) in stimulating cells in their late stage of osteoblastic differentiation [36].

The influence of hormones and local peptidic factors on cell metabolism is often regulated by enzymatic activity of cell surface peptidases such as neprilysin (NEP) [50]. NEP is a member of a family of cell surface zinc metallopeptidases that shows preference for small peptide substrates and is associated with the degradation of several bioactive peptides in vivo [51]. This peptidase was found to be present on all bone-forming cells, but was more highly expressed in the bones of young animals. Several osteogenic peptides are good NEP substrates in vitro, including OGP, which can be cleaved and presumably inactivated by NEP at Gln^5^-Gly^6^ or Thr^8^-Leu^9^ bonds.

### 2.2. Effect of OGP and OGP(10–14) on Cells Functions in the Context of Bone Regeneration

The osteogenic properties of OGP and OGP(10–14) have been most commonly evaluated in in vitro studies using cellular systems based on osteoblastic cell lines or marrow stromal cells of either murine or human origin (Table 2). These studies showed that both native and synthetic OGP (sOGP) stimulated proliferation and activity of osteoblastic MC3T3-E1, ROS 17/2.8 cells and NIH 3T3 fibroblasts [33,35,43,47].

The ideal concentration of OGP to stimulate the proliferation and activity of osteoblasts was found in the MC3T3-E1 in which cell number was increased in peaks at peptide concentrations of 10^−13^ M, indicating a considerably higher sensitivity of these cells to OGP peptide [43]. The alkaline phosphatase activity (ALP) was also modestly inhibited at peptide concentrations of 10^−13^ M [33]. Similar to OGP peptide, different concentrations (10^−13^ up to 10^−8^ M) of OGP(10–14) were evaluated in primary human osteoblast (hOB) cells [52]. In hOB cells, a maximum concentration of 10^−9^ M OGP(10–14) stimulated bone formation and mineralization through osteocalcin synthesis and phosphatase activity. However, the ideal concentration of OGP(10–14) to stimulate the proliferation of hOB cells was 10^−12^ M. In addition, OGP(10–14) inhibited apoptosis and prevented the decrease in osteoprotegerin secretion induced by exposure of hOB cultures to dexamethasone, with a maximal effect at 10^−9^ M and 10^−12^ M OGP(10–14) concentrations, respectively. These results suggest the use of the OGP(10–14) as a possible anabolic treatment for osteoporosis [52].

Furthermore, OGP and OGP(10–14) are also potent regulators of marrow stromal cells, wherein the main OGP activity in marrow systems is a marked stimulation of ALP activity and matrix mineralization [37,52,53,54,55,56,57]. Compared with other growth factors such as growth hormone (GH) and bFGF, OGP is the most potent enhancer of ALP activity and mineralization in marrow stromal cells [53]. Similarly, OGP(10–14) positively regulates bone formation by stimulating the differentiation of rat mesenchymal stem cells(MSCs) into osteoblasts and concurrently inhibiting adipocyte formation [37].

### 2.3. Potential Biological Mechanisms of OGP and OGP(10–14) in Proliferation and Differentiation of Osteoblasts

Although the exact molecular mechanisms governing osteoblastic proliferation and differentiation are not completely known, some signaling cascades involved in these processes have been investigated [55,56,57].

**Figure 2:**
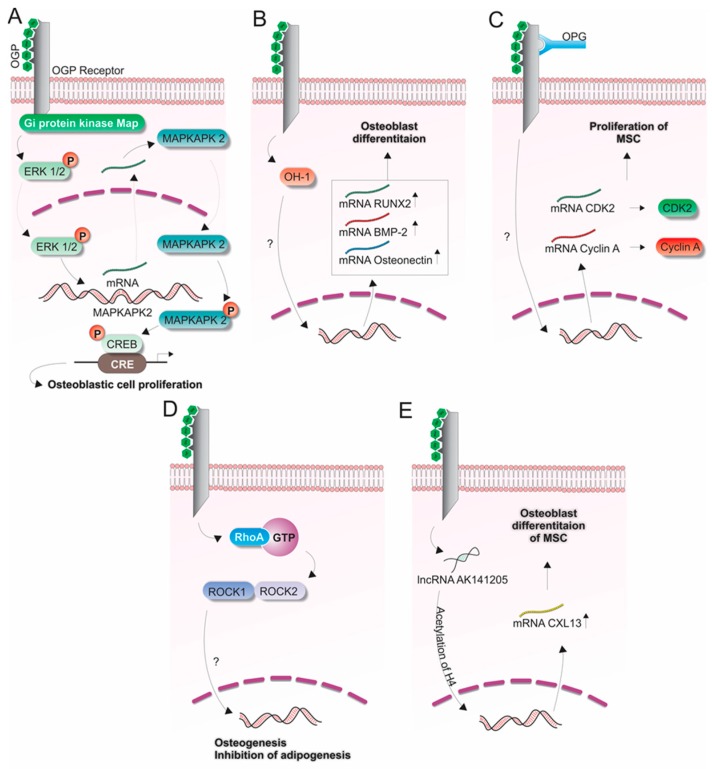
Signaling pathways potentially associated with OGP(10–14) (in green) during the bone formation process. (**A**) Proliferation of osteoblastic cells (MC3T3-E1) induced by OGP involving the ERK1/2, MAPKAPK2, CREB signaling cascade [58]; (**B**–**D**) Differentiation of mesenchymal stem cells (MSCs) to osteoblasts by linking the OGP(10–14) to its cellular receptor. (**B**) Participation of heme oxidase 1 (HO-1), Osteonectin, BMP-2 and RUNX-2 [56]; (**C**) Extracellular osteoprotegerin (OPG) influencing the cytoplasmic expression of Cyclin A and CDK2 [55]; (**D**) RhoA/ROCK signaling cascade in the presence of GTP [57]; (**E**) Participation of lncRNA AK141205 in the acetylation of H4 influencing the expression of CXCL13 in osteoblast differentiation [60].

In MC3T3-E1 osteoblastic cells, OGP(10–14) showed a mitogenic effect involving the rapid phosphorylation of Extracellular signal–regulated kinases 1/2 (ERK1/2), and de novo Mitogen-activated protein kinase-activated protein kinase 2 (MAPKAPK2) mRNA and protein synthesis. After phosphorylation of ERK1/2 via activation of G_i_ protein kinase mitogen-activated protein (MAP) by OGP(10–14), an increased expression and activation of MAPKAPK2 phosphorylation and increased transcriptional activity of the cAMP response element-binding (CREB) transcription factor were observed, which resulted in cell proliferation [58] (Figure 2A).

Based on this positive role of OGP and OGP(10–14) in multipotent cells, some studies have investigated the mechanisms whereby OGP regulates the differentiation of MSCs into osteoblasts [55,56,57]. In this context, Vanella et al. [56] investigated the heme oxidase 1 (HO-1) protein and found that its expression was increased during differentiation of osteoblasts from MSCs. Interestingly, OGP induced HO-1 expression and enhanced ALP activity and gene expression of Bone Morphogenetic Protein 2 (BMP2), Osteonectin and Runt-related transcription factor 2 (RUNX2) (Figure 2B). In addition, after exposure of MSCs to high glucose, OGP reversed the decrease in osteocalcin and osteoprotegerin levels by inducing HO-1 expression. Similarly, decreased levels of 5′-AMP-activated protein kinase (pAMPK), serine/threonine protein kinase (pAKT) and nitric oxide synthase (eNOS) phosphorylation, due to a glucose-induced reduction in HO-1 expression, could be reversed by OGP. Moreover, significant increases in ALP activity and collagen accumulation were observed in rabbit MSCs after transfection with a vector containing a DNA fragment of OGP [54]. The ability of OGP to promote the differentiation of MSCs into osteoblast cells, combined with the relatively low molecular weight of OGP, made it suitable for gene therapy for bone fracture and osteoporosis.

Fei et al. [55] found that OGP stimulates the proliferation of MSCs derived from OPG-deficient mice (OPG, osteoprotegerin) by triggering the Cyclin-dependent kinase–cyclin A (CDK2–cyclin A) pathway, since they observed expression of CDK2 and cyclin A, both at the mRNA and protein levels. OGP might also interact with OPG once the absence of OPG might cut down the effect of OGP on MSC osteoblastic differentiation (Figure 2C).

During the process of cell differentiation, the membrane expression of the active form of Ras Homolog Gene Family Member (RhoA) in MSCs was increased, leading to investigation of the RhoA/Rho-associated protein kinase (ROCK) signaling. RhoA becomes activated after binding to the GTP protein. Subsequently, ROCK 1 and 2 are the major proteins associated with RhoA kinase (Figure 2D).

Increased binding of RhoA to the GTP protein was observed in human MSC cells treated with OGP [57]. Moreover, when ROCK inhibitors were used, a reduction in osteoblast differentiation could be observed after the stimulation of MSCs with OGP. Therefore, it was suggested that OGP promotes osteogenesis, while adipogenesis is inhibited by RhoA/ROCK signaling (Figure 2D).

Additionally, the molecular mechanism of OGP, as a regulator of osteogenic differentiation of MSCs, has revealed that AK141205 (a long non-coding RNA, lncRNA, whose expression is induced by OGP) may enhance the expression of C-X-C motif ligand 13 (CXCL13) resulting in osteogenic differentiation [60] (Figure 2E). The inhibition of AK141205, using a RNA interference (si-AK141205), suppressed the OGP-induced formation of calcium salt nodules, ALP activity and the expression of genes associated with osteogenic differentiation. Moreover, analysis of CXCL13 expression, a chemokine essential for functional maintenance of osteoblasts [61,62], revealed that AK141205 positively promoted CXCL13 expression via acetylation of H4 histone in the promoter region. In this way, this study suggested a novel role for AK141205 and CXCL13 as regulators of OGP-induced osteogenic differentiation of MSCs.

### 2.4. Medical Applications of OGP and OGP(10–14) in Bone Repair/Regeneration

The in vivo effect of exogenously administered OGP in bone formation was first described by Bab et al. [33]. In this study, OGP was intravenously administered to adult male rats for 9 consecutive days and enhanced bone formation associated with increased trabecular bone mass was observed in the mandibular condyles. After this study, the use of intravenously/subcutaneously administrated OGP was extensively evaluated in models of fracture repair in rats (Table 3). In general, these studies have shown faster healing associated with increased bone formation in the OGP treated fractures. Moreover, OGP-treated animals showed higher mechanical toughness of the fracture callus [63]. Interestingly, Brager et al. [38] aspirated the bone marrow from rat femurs and showed a large increase in mitogenicity and osteogenicity in the marrow-derived cultures from rats treated with OGP. These combined results suggest that OGP could be utilized as a potential therapy for the acceleration of bone regeneration in cases of fracture repair/regeneration and perhaps other bone injuries [63].

Similarly, using a different model of bone healing, Zhao et al. [64] evaluated the potential of systemically-administered OGP during distraction osteogenesis in rabbit tibia. Distraction osteogenesis is a valuable treatment method that allows limb lengthening or reconstruction of large bone defects. However, a long period is required for consolidation of a distraction callus [65]. The use of OGP in distraction osteogenesis therapy enhanced new bone formation, and showed increased bone fill and greater torsional stiffness in comparison with distraction osteogenesis alone. In this way, systemically-administered OGP may shorten the duration of the intervention and decrease the prevalence of complications in distraction osteogenesis therapy.

The use of OGP and OGP(10–14) has also been proposed in osteoporosis treatment [40,52]. Chen et al. [40] evaluated the effect of systemic administration of this peptide in the reversal of ovariectomy-induced bone loss. This study demonstrated that OGP reversed the majority of trabecular bone loss in the proximal tibial metaphysis of ovariectomized mice. Furthermore, OGP(10–14) had an even stronger osteogenic effect than OGP.

Based on the positive effects of systemically-administered OGP and OGP(10–14), the peptides were considered good candidates for bone tissue engineering applications. Therefore, studies have been carried out to evaluate the effectiveness of OGP and OGP(10–14) in tissue-engineered scaffolds/membranes/hydrogels (Table 3). Shuqiang et al. [66] evaluated the effect of locally applied OGP incorporated into poly(lactic-*co*-glycolic acid) (PLGA) scaffolds in comparison with systemically administered OGP in the healing of segmental long bone defects in rabbits. In this study, 100 µg of OGP were incorporated by adsorption into each PLGA scaffolds. Histomorphometric and radiographic analysis showed that bone formation and union were significantly higher after local application of the OGP than after systemic administration. Moreover, degradation of the scaffold material occurred while new bone tissue was formed. Based on these results, the PLGA porous scaffold associated with OGP could be considered a therapeutic alternative to the use of autologous or allogeneic bone grafts in orthopedic surgery.

In dentistry, OGP has been incorporated into regenerative membranes used in the Guided Tissue Regeneration (GTR) technique. GTR is a therapy that utilizes membranes as biological barriers to prevent subepithelial connective tissue invagination into intrabony defects, preserving the space for bone neoformation [67]. Saska et al. [68] developed a bacterial cellulose (BC) membrane that associated with OGP and OGP(10–14) peptides. In vitro assays demonstrated no cytotoxic, genotoxic or mutagenic effects of BC membranes. Cell viability/proliferation, total protein content, ALP activity and mineralization assays indicated that BC-OGP membranes enabled the highest development of the osteoblastic phenotype in vitro. Moreover, these membranes associated with hydroxyapatite (BC-HA) with OGP or OGP(10–14) were evaluated in critical-size calvarial defects in mice [69]. In both studies, the OGP and OGP(10–14) were incorporate by adsorption at concentration of 10^−9^ M. The BC-HA membranes promoted better bone formation in critical-size mice calvarial defects. However, the analysis of gene expression showed that the BC-HA-OGP/OGP(10–14) membranes promoted an acceleration of osteoblast differentiation/activity in the early periods of analysis. Nevertheless, the incorporation of the peptides at concentrations of 10^−9^ M did not improve bone regeneration potential in long-term analysis. These results suggest that material modifications are needed to improve the effectiveness of the peptides release in the required concentration for longer periods [69].

Stakleff et al. [72] developed a novel class of amino acid-based poly(ester urea)s (PEU) materials which are biodegradable in vivo and present mechanical properties superior to conventionally used polyesters. Additionally, the use of OGP(10–14) as a covalent crosslinker for the PEU materials was evaluated in vitro and in a subcutaneous animal model. The polymer chains were photo-chemically crosslinked with 1% OGP and the results showed that OGP could act as a bioactive crosslinking agent inasmuch as the materials were biocompatible, showing no cytotoxicity and enhanced proliferative activity of MC3T3-E1 osteoblasts. The in vivo results indicated that polymers containing 0.5% and 1.0% OGP exhibited a significantly favorable interaction with the amount of biodegradation and incorporation of tissue into the polymer materials compared to other polymers without OGP. Therefore, these materials have the potential to improve regeneration of bone defects. Otherwise, tests in bone repair model need to be executed.

In order to specifically induce the osteogenic differentiation of human MSCs (hMSCs), a multifunctional injectable vehicle for the co-delivery of hMSCs and osteoinductive peptides was proposed by Maia et al. [71]. Hydrogels showed great potential as cell vehicles for minimally invasive bone regeneration therapies. These materials form highly hydrophilic 3D networks that recreate some features of native extracellular matrices providing adequate cellular microenvironments for cell differentiation and proliferation [73]. The local release of the bioactive portion of OGP occurs via proteolytic cleavage of the peptide linkers by metalloproteinase 2. For this, both proteins were chemically grafted to the natural polysaccharide alginate, providing localized OGP delivery for variable time periods and keeping this peptide in close proximity to the targeted host cells at the injury site. More specifically, aqueous carbodiimide chemistry was used to connect peptide N-terminal amino groups to alginate carboxyl groups via a peptide bond. Moreover, after the addition of hMSCs to the hydrogel, OGP specifically guides cells towards differentiation towards the osteoblastic lineage. After using the OGP–alginate hydrogel in an ectopic setting, hMSCs were able to proliferate, migrate out of the hydrogels and produce and mineralize an endogenous extracellular matrix. In this approach, OGP–alginate stimulated hMSC osteogenesis and represents an alternative therapy for minimally invasive healing of small bone defects [71].

Policastro et al. [45] successfully developed non-functionalized and functionalized phenylalanine-based poly(ester urea) porous scaffolds with various pore sizes. The phenylalanine PEUs (poly(1-Phe-6)) were modified by tethering osteogenic growth peptide (OGP) to tyrosine-based monomer subunits. Then, OGP-tethered PEUs have been fabricated into porous scaffolds and cultured in vitro to examine their effect on differentiation hMSCs towards the osteogenic lineage. In vitro analysis showed that the addition of OGP to the scaffolds resulted in faster osteoblast differentiation than the unfunctionalized scaffolds. Furthermore, scaffolds with large pores allowed greater osteogenic gene expression by MSCs than controls and small pore sizes. The authors suggested that large pore sizes could be necessary for bone differentiation to ensure cell penetration into the scaffold.

## 3. Conclusions

The identification of new approaches to stimulate osteoblast function and bone formation is critical to improve the treatment of fractures and other bone diseases and ultimately improve quality of life for patients. For this purpose, extensive investigations of the signaling pathways involved in the influence of OGP and OGP(10–14) peptides on osteoblast activity are still necessary. This review discussed about some studies that investigated the signaling and interaction between these peptides and other molecules during osteoblast proliferation and differentiation. Therefore, new discoveries in this direction may result in new approaches that involve combining different molecules that interact with these peptides in order to improve their effectiveness in bone repair/regeneration. Therefore, further investigation studies about OGP and OGP(10–14) peptides are necessary to improve and extensively enable the clinical use of these peptides in humans for medicine regenerative.

## Figures and Tables

**Table 1:** Growth factors associated with osteoblast function.

Growth Factor	Abbreviation	OMIM	Function	References
Fibroblast Growth Factor 2	FGF 2	134920	Stimulates osteoblast proliferation and decreases differentiation markers such as alkaline phosphatase and type I collagen	[5]
Fibroblast Growth Factor 6	FGF 6	134921	Increases human primary osteoblasts proliferation and reduces their differentiation	[6]
Fibroblast Growth Factor 8	FGF 8	600483	Increases osteoblast proliferation and alkaline phosphatase production and bone formation at an early stage of osteoblastic differentiation	[7]
Fibroblast Growth Factor 18	FGF 18	603726	Stimulates osteoblast proliferation and inhibits their differentiation and matrix synthesis in a dose-dependent manner	[8]
Transforming Growth Factor β-1	TGF β-1	190180	Stimulates or inhibits the osteogenic differentiation of bone marrow stromal cells	[9,10]
Insulin-like Growth Factor 1	IGF 1	147440	Promotes osteoblast differentiation, proliferation and mineralization in vitro	[11]
Platelet-derived Growth Factor	PDGF	190040	Induces osteoblastic cell migration and proliferation	[12]
Bone Morphogenetic Protein 2	BMP 2	112261	Induces osteoblast differentiation and stimulates the expression of mineralization-associated genes	[13]
Bone Morphogenetic Protein 4	BMP 4	112262	Induces differentiation of osteoblast-like cells	[14]
Bone Morphogenetic Protein 6	BMP 6	112266	Stimulates osteoblast differentiation and mineralization	[15]
Bone Morphogenetic Protein 7	BMP 7	112267	Induces mesenchymal stem cells to differentiate into osteoblasts in vitro.	[16]
Vascular Endothelial Growth Factor	VEGF	192240	Enhances in vitro osteogenic proliferation and differentiation	[17]
Epidermal Growth Factor	EGF	131530	Up-regulates osteoblast proliferation and osteoblastic markers and inhibits bone nodule formation	[18]
Connective Tissue Growth Factor	CTGF	121009	Promotes the proliferation and differentiation of osteoblasts	[19]
Mechano-growth Factor	MGF	Not found	Stimulates osteoblast proliferation and inhibits their differentiation and mineralization	[20]
Activin A	Activin A	147290	Inhibits early differentiation of osteoblasts	[21]
Twist-related protein 1	TWIST1	607556	Inhibits osteoblast differentiation	[22]
Hepatocyte Growth Factor	HGF	142409	Stimulates osteoblasts proliferation and differentiation	[23,24]
Growth Differentiation Factor 5	GDF-5	601146	Stimulates early osteoblast differentiation and extracellular matrix production	[25]
Cartilage Oligomeric Matrix Protein	COMP	600310	Promotes mesenchymal stem cells differentiation to chondrocytes and osteoblasts	[26]
Preadipocyte factor-1	DLK1/Pref-1	176290	Inhibits the formation of mature osteoblasts	[27]
Wnt-inducible signaling pathway protein 1	WISP-1	603398	Influences on bone cell differentiation and function by enhancing the effects of BMP-2	[28]
Osteoclast Inhibitory Lectin	OCIL	Not found	Inhibits osteoblast differentiation and function in vitro	[29]
Endothelin 1	EDN1/ET 1	131240	Mediates osteoblastic bone metastases by stimulating osteoblast proliferation and new bone formation	[30]
Lactotransferrin/Lactoferrin	LTF	150210	Promotes primary osteoblast proliferation and differentiation via up-regulation of IGF-1 expression	[31]

OMIM: Online Mendelian Inheritance in Man [32].

**Table 2:** OGP and OGP(10–14) in vitro studies.

Peptide	Cell lineage	Biological Activity	Reference
OGP	ROS 17/2.8 cells; osteoblastic MC3T3-E1 cells; fibroblastic NIH 3T3 cells	Stimulates proliferation and alkaline phosphatase activity in osteogenic/fibroblastic cell lines in vitro	[33]
OGP	Osteoblastic MC3T3-E1 cells and fibroblastic NIH 3T3 cells	Stimulates proliferation in osteoblastic/fibroblastic cell lines in vitro	[35]
OGP	Marrow stromal cells from human and rabbit	Acts as a potent regulator of marrow stromal cells, enhancing cell proliferation, phosphatase activity and matrix mineralization	[53]
OGP	Osteoblastic MC3T3-E1 cells	OGP binds to both native and activated human plasma α2-macroglobulin (α2M). Native α2M substantially increased the OGP proliferative effect in osteoblastic cells. The activated α2M inhibited the osteoblastic proliferation induced by OGP	[44]
OGP	Osteoblastic MC3T3-E1 cells and fibroblastic NIH 3T3 cells	(a) Two OGP binding protein (OGPBP) complexes were identified; (b) the OGP production is up- and downregulated by low and high doses of exogenous OGP, respectively; (c) For proliferative evaluation, osteoblastic cell lines were more sensitive to OGP than nonosteoblastic cell systems	[47]
OGP and OGP(10–14)	Osteoblastic MC3T3-E1 cells	Isolation of active C-terminal truncated pentapeptide of OGP, OGP(10–14), and their mitogenic potential at osteoblastic cells	[48]
OGP(10–14)	Osteoblastic MC3T3-E1 cells and fibroblastic NIH 3T3 cells	Increase of osteoblastic/fibroblastic cell lines proliferative activity induced by OGP(10–14)	[40]
OGP and OGP(10–14)	Osteoblastic MC3T3-E1 cells	OGP(10–14) induces the proliferative activity of osteoblastic cells by mitogenic Gi protein MAP kinase-signaling cascade (Figure 2A)	[34]
OGP(10–14)	Osteoblastic MC3T3-E1 cells	Proliferative activity signaling cascade: ERK1/2 stimulation by OGP(10–14) increases the de novo MAPKAPK2 synthesis resulting in CREB phosphorylation and enhances of transcriptional activity (Figure 2A)	[58]
OGP(10–14)	Primary human osteoblasts (hOB)	OGP(10–14) inhibits hOB apoptosis induced by glucocorticoid (GC) and increase OPG secretion restoring the altered expression of OPG induced by GCs to physiological levels	[52]
OGP(10–14)	Primary bone marrow-derived ratmesenchymal stem cells (MSCs)	OGP(10–14) promoted osteogenic differentiation of MSCs and concurrently inhibited adipocyte formation	[37]
OGP	Primary bone marrow-derived MSCs from osteoprotegerin-deficient mice	OGP stimulating MSC proliferation in OPG-deficient mice by CDK2/cyclin A pathway (Figure 2C)	[55]
OGP	Human bone marrow-derived MSCs	Exposure of MSC to high glucose levels decreased bone-related protein expression, which was reversed by OGP-mediate increase in HO-1 expression (Figure 2B)	[56]
OGP	Human bone marrow-derived MSCs	OGP stimulates MSC differentiation via the activation of RhoA/ROCK pathway (Figure 2D)	[59]
OGP	Primary bone marrow-derived rabbit MSCs	ALP activity increased and enhanced collagen accumulation in OGP gene-transfected MSCs	[54]
OGP	Primary bone marrow-derived rabbit MSCs	MSC stimulation with OGP induced upregulation of AK141205 and CXCL13 and osteogenic differentiation	[60]

MAP: Mitogen-activated protein; ERK: Extracellular signal–regulated kinases; MAPKAPK: Mitogen-activated protein kinase-activated protein kinase 2; CDK: cyclin-dependent kinase; HO: heme oxidase; RhoA: Ras Homolog Gene Family Member, A; ROCK: Rho-associated protein kinase; ALP: alkaline phosphatase; AK141205: a long non-coding RNA; CXCL13: C-X-C motif ligand 13.

**Table 3:** Medical applications of OGP and OGP(10–14) peptides.

Peptide	Application	Model	Defects	Reference
OGP	Intravenous administration	Male rats	Increased trabecular bone mass in the mandibular condyles	[33]
OGP	Intravenous administration	Male rabbits	The callus formation and cortical healing is enhanced by OGP treatment in tibiae fracture	[39]
OGP	Intravenous administration	Ovariectomized female mice	Reverses the trabecular bone loss in ovariectomized mice	[40]
OGP	Subcutaneous administration	Male rats	Promotes an earlier bone-repair callus in femoral fracture	[38]
OGP	Intravenous administration	Male rats	Improved callus formation and function in femoral fracture	[63]
OGP	Poly (lactic-*co*-glycolic) acid scaffolds	Male rabbits	Accelerates healing of segmental radius bone defects	[66]
OGP	Intravenous administration	Male rabbits	Promotes optimal new bone formation during distraction osteogenesis	[64]
OGP	Calcium phosphate thin films	Not Applied	Not Applied	[59]
OGP and OGP(10–14)	Bacterial Cellulose Membrane	CHO-K1 and osteoblastic cells	No cytotoxic, genotoxic or mutagenic effects of BC membranes	[68]
OGP(10–14)	Poly(ester urea) homopolymers	hMSCs and Male rats	Significant tissue-scaffold integration and promotion of osteogenesis/angiogenesis	[45]
OGP	Mesoporous silica and mesoporous silica/apatite	Not Applied	Not Applied	[70]
OGP(10–14)	Alginate hydrogels	Immunodeficient male mice	OGP increases the hydrogels degradation and the vascularized connective tissue colonization evaluated by subcutaneous implantation	[71]
OGP and OGP(10–14)	Bacterial cellulose-hydroxiapatite membrane	Male mice	Enhances bone formation in critical-size calvarial defects in mice mainly in early stages of bone regeneration	[69]

## Supplementary Materials

Supplementary materials can be found at www.mdpi.com/1422-0067/17/11/1885/s1.
