# Supplementary material for: Role of Osteogenic Growth Peptide (OGP) and OGP(10–14) in Bone Regeneration: A Review
Source: Int J Mol Sci. 2016 Nov 22;17(11):1885. doi: 10.3390/ijms17111885 (PMC5133884; doi:10.3390/ijms17111885)
Supplement: Supplementary file 1 [file ijms-17-01885-s001.pdf]

Supplementary Materials: Role of Osteogenic Growth Peptide (OGP) and OGP(10–14) in Bone Regeneration: A Review

Suzane C. Pigossi, Marcell C. Medeiros, Sybele Saska, Joni A. Cirelli and Raquel M. Scarel-Caminaga

1. Introduction

As demonstrated in Table 1, there are many growth factors related to bone repair/regeneration. Bone morphogenetic proteins (BMPs) have been subject to intense research for their possibly therapeutic use in applications in bone repair/regeneration [1]. At least 20 types of BMPs have been identified in humans among them, BMP-2, 4, 5, 6, and 7 have demonstrated strong osteogenic capacity [2]. In mesenchymal stem cells (MSCs), BMP-2 increases osteocalcin levels in vitro [3] and induces bone formation in vivo [4]. Recombinant forms of BMPs, particularly rhBMP-2 (Infuse, Medtronic Sofamor Danek, Memphis, TN, USA), are commercially available for clinical application in treating bone defects [5].

The insulin–like growth factors (IGFs) are another abundant growth factor produced by osteoblasts and present in bone [6]. These molecules are important regulators of osteoblast functions and are required for optimal bone development and maintenance. IGF-1 promotes osteoblast proliferation, differentiation and mineralization in primary calvarial osteoblasts in vitro [7].

Similarly, fibroblast growth factors (FGFs) are polypeptides that regulate several important cellular processes and play a critical role in bone formation [8]. Among them, FGF-2 is known to be an important regulator of osteoblastic activity, since it stimulates osteoblast replication and decreases the expression of differentiation markers by this cell in vitro [9,10]. FGF-2 is also a critical determinant of bone mass in mice by modulating the Wnt signaling pathway [11].

Other biological mediators show similar biological activities in bone, such as connective tissue growth factor (CTGF), hepatocyte growth factor (HGF), growth differentiation factor 5 (GDF-5) and epidermal growth factor (EGF). In general, these mediators act by upregulating osteoblast proliferation and/or differentiation during bone neoformation [12–14]. On the other hand, activin A, osteoclast inhibitory lectin (OCIL), preadipocyte factor-1 (DLK1/ Pref-1) and twist-related protein 1 (TWIST1) are negative regulators of osteoblast differentiation in bone remodeling [15–18].

2. Methods

This study Review was developed utilizing electronic searches of the PubMed database which were conducted in April 2016 for publications that investigated the role of OGP and OGP(10–14) in bone regeneration. Relevant papers were identified through a search of this database using the following terms: "Osteogenic Growth Peptide" or "OGP" or "OGP(10–14)" alone or associated with "bone regeneration". A literature search was conducted using the Endnote Program™ X7 version (Thomson Reuters, New York, NY, USA) in order to eliminate duplicate references.

Three hundred and ninety eight English publications were found. After the publication selection, 47 articles were related to the investigation of OGP/OGP(10–14). Among these, 35 references investigated the role of OGP and/or OGP(10–14) in bone regeneration, 9 references investigated the role of OGP and/or OGP(10–14) in hematopoiesis and 3 references were reviews. In the present literature review was considered the 35 publications that characterized and investigated the role of these peptides (alone or in association with biomaterials) in bone repair/regeneration.

References

1. Gautschi, O.P.; Frey, S.P.; Zellweger, R. Bone morphogenetic proteins in clinical applications. *ANZ J. Surg.* **2007**, *77*, 626–631.
2. Chen, G.; Deng, C.; Li, Y.P. TGF-beta and BMP signaling in osteoblast differentiation and bone formation. *Int. J. Biol. Sci.* **2012**, *8*, 272–288.
3. Huang, Z.; Ren, P.G.; Ma, T.; Smith, R.L.; Goodman, S.B. Modulating osteogenesis of mesenchymal stem cells by modifying growth factor availability. *Cytokine* **2010**, *51*, 305–310.
4. Noel, D.; Gazit, D.; Bouquet, C.; Apparailly, F.; Bony, C.; Plence, P.; Millet, V.; Turgeman, G.; Perricaudet, M.; Sany, J.; Jorgensen, C. Short-term BMP-2 expression is sufficient for in vivo osteochondral differentiation of mesenchymal stem cells. *Stem Cells* **2004**, *22*, 74–85.
5. De Freitas, R.M.; Susin, C.; Spin-Neto, R.; Marcantonio, C.; Wikesjo, U.M.; Pereira, L.A.; Marcantonio, E., Jr. Horizontal ridge augmentation of the atrophic anterior maxilla using rhBMP-2/ACS or autogenous bone grafts: A proof-of-concept randomized clinical trial. *J. Clin. Periodontol.* **2013**, *40*, 968–975.
6. Mohan, S.; Baylink, D.J. Insulin-like growth factor system components and the coupling of bone formation to resorption. *Hormone Res.* **1996,** *45* (Suppl. 1), 59–62.
7. Govoni, K.E. Insulin-like growth factor-I molecular pathways in osteoblasts: Potential targets for pharmacological manipulation. *Curr. Mol. Pharmacol.* **2012**, *5*, 143–152.
8. Marie, P.J. Fibroblast growth factor signaling controlling bone formation: An update. *Gene* **2012**, *498*, 1–4.
9. Rodan, S.B.; Wesolowski, G.; Yoon, K.; Rodan, G.A. Opposing effects of fibroblast growth factor and pertussis toxin on alkaline phosphatase, osteopontin, osteocalcin, and type I collagen mRNA levels in ROS 17/2.8 cells. *J. Biol. Chem.* **1989**, *264*, 19934–19941.
10. Hurley, M.M.; Abreu, C.; Harrison, J.R.; Lichtler, A.C.; Raisz, L.G.; Kream, B.E. Basic fibroblast growth factor inhibits type I collagen gene expression in osteoblastic MC3T3-E1 cells. *J. Biol. Chem.* **1993**, *268*, 5588–5593.
11. Fei, Y.; Xiao, L.; Doetschman, T.; Coffin, D.J.; Hurley, M.M. Fibroblast growth factor 2 stimulation of osteoblast differentiation and bone formation is mediated by modulation of the Wnt signaling pathway. *J. Biol. Chem.* **2011,** *286*, 40575–40583.
12. Kawaki, H.; Kubota, S.; Suzuki, A.; Suzuki, M.; Kohsaka, K.; Hoshi, K.; Fujii, T.; Lazar, N.; Ohgawara, T.; Maeda, T.; et al. Differential roles of CCN family proteins during osteoblast differentiation: Involvement of Smad and MAPK signaling pathways. *Bone* **2011**, *49*, 975–989.
13. Koch, F.P.; Weinbach, C.; Hustert, E.; Al-Nawas, B.; Wagner, W. GDF-5 and BMP-2 regulate bone cell differentiation by gene expression of MSX1, MSX2, Dlx5, and Runx2 and influence OCN gene expression in vitro. *Int. J. Periodontics Restor. Dent.* **2012**, *32*, 285–293.
14. Laflamme, C.; Curt, S.; Rouabhia, M. Epidermal growth factor and bone morphogenetic proteins upregulate osteoblast proliferation and osteoblastic markers and inhibit bone nodule formation. *Arch. Oral Biol.* **2010**, *55*, 689–701.
15. Ikenoue, T.; Jingushi, S.; Urabe, K.; Okazaki, K.; Iwamoto, Y. Inhibitory effects of activin-A on osteoblast differentiation during cultures of fetal rat calvarial cells. *J. Cell. Biochem.* **1999**, *75*, 206–214.
16. Nakamura, A.; Ly, C.; Cipetic, M.; Sims, N.A.; Vieusseux, J.; Kartsogiannis, V.; Bouralexis, S.; Saleh, H.; Zhou, H.; Price, J.T.; et al. Osteoclast inhibitory lectin (OCIL) inhibits osteoblast differentiation and function in vitro. *Bone* **2007**, *40*, 305–315.
17. Abdallah, B.M.; Jensen, C.H.; Gutierrez, G.; Leslie, R.G.; Jensen, T.G.; Kassem, M. Regulation of human skeletal stem cells differentiation by Dlk1/Pref-1. *J. Bone Miner. Res.* **2004**, *19*, 841–852.
18. Komaki, M.; Karakida, T.; Abe, M.; Oida, S.; Mimori, K.; Iwasaki, K.; Noguchi, K.; Oda, S.; Ishikawa, I. Twist negatively regulates osteoblastic differentiation in human periodontal ligament cells. *J. Cell. Biochem.* **2007**, *100*, 303–314.
